# Follow up Testing Among Male U.S. Air Force Basic Trainees Diagnosed with Chlamydia or Gonorrhea, 2017–2023

**Published:** 2026-01-26

**Authors:** Rachel E. Powers, Erin L. Winkler, Theresa M. Casey, Angela B. Osuna, Ga On Jung, Joseph E. Marcus

**Affiliations:** Department of Medicine, Brooke Army Medical Center, Joint Base San Antonio–Fort Sam Houston, San Antonio, TX: Capt Powers, Maj Marcus; Trainee Health Surveillance, 559th Medical Group, Joint Base San Antonio-Lackland, TX: Lt Col Winkler, Brig Gen (ret) Casey, Ms. Osuna, Ms. Jung; Department of Medicine, Uniformed Services University of Health Sciences and Infectious Diseases Service, Brooke Army Medical Center: Maj Marcus

## Abstract

While female U.S. Air Force and Space Force basic military trainees are screened universally for gonorrhea and chlamydia, male basic trainees are tested only when symptomatic or upon patient request. Epidemiology and follow-up testing of male basic trainees who test positive for gonorrhea or chlamydia in training is unclear. All active duty male basic trainees at Joint Base San Antonio–Lackland who tested positive for gonorrhea or chlamydia from 2017 through 2023 (50 of 182,726 total male trainees, 0.03%) were matched, 1-to-1, by age and accession date, with active duty female basic trainees who tested positive for the same pathogen. Medical records from military hospitals and clinics were reviewed for follow-up testing within 12 months of the initial positive test and subsequent diagnoses for chlamydia and gonorrhea up to 3 years afterwards, or July 1, 2024, whichever occurred first. Among 50 male basic trainees, 30 (60%) reported symptoms when presenting for testing. Most cases (86%) were due to chlamydia. Only 56% (n=28) of male trainees had follow-up testing within 1 year, compared to 76% (n=38) of matched female basic trainees (OR 0.4, 95% CI: 0.17, 0.95). Low screening for chlamydia and gonorrhea among male basic trainees may contribute to reduced follow-up testing and represents a missed opportunity to identify infections, prevent transmission, and reduce the burden of infection in this population.

**What are the new findings?**

Male basic military trainees who tested positive for gonorrhea or chlamydia had follow-up testing rates significantly below guideline recommendations. Rates of future infections among male basic trainees were not, however, statistically lower than female trainee rates of future infections.

**What is the impact on readiness and force health protection?**

These findings support universal gonorrhea and chlamydia screening for male trainees at higher risk for infection to reduce the impact of untreated infections on military readiness for individuals and their partners, in addition to facilitating provision of available methods of sexually transmitted infection prevention.

Service in the U.S. military has been associated with increased risk of sexually transmitted infections (STIs) such as gonorrhea and chlamydia. ^1^ Before basic military training (BMT), all potential enlistees undergo medical evaluation including HIV testing, to ensure they meet criteria for accession, but they are not tested for chlamydia ^2 - 6^ or gonorrhea. BMT is an 8-week training program that is the sole point for civilian entry into the enlisted ranks of the U.S. Air Force and Space Force. During BMT, all trainees have access to universal, no-cost health care at both primary care clinics and emergency care facilities on base. ^7^ Because male BMT trainees are not screened for gonorrhea or chlamydia, they are only tested if they are symptomatic or request testing.

A recent study of universally screened male Air Force BMT trainees found similar overall rates of chlamydia with female Air Force BMT trainees, although most infections were asymptomatic. ^8^ Women entering U.S. Air Force and Space Force BMT are universally screened for chlamydia and gonorrhea due to known long-term sequelae of untreated infections, previously documented high rates of positivity, and guidelines recommending universal female screening. Positivity rates among female BMT trainees are approximately 0.3% for gonorrhea and 5.0% for chlamydia. With the exception of the Army, all services require universal screening for female BMT trainees. ^7^

Current U.S. Centers for Disease Control and Prevention (CDC) guidelines recommend testing for re-infections 3 months after a gonorrhea or chlamydia diagnosis, regardless of patient sex or risk factors for future infection. ^5^ Additionally, guidelines recommend that men at high risk for STIs, such as men who have sex with men, should be screened at least annually for chlamydia and gonorrhea. Annual chlamydia screening is required by all services for female service members under age 25 years. ^7^

While the screening disparity between male and female BMT trainees is evident, it is unclear how this may affect future testing and STI diagnoses for male trainees who test positive for chlamydia or gonorrhea during BMT. Previously evaluated 2006-2021 data from 5,022 female BMT trainees who tested positive for gonorrhea or chlamydia showed a high follow-up testing rate (69.7%) within 1 year, as well as a relatively high rate (15.9%) of positivity upon repeat testing. ^2^ This study investigated the incidence of gonorrhea and chlamydia in male Air Force and Space Force BMT trainees from 2017 through 2023 and compared follow-up testing and clinical outcomes with female BMT trainees.

## Methods

This retrospective matched cohort study evaluated all active duty male BMT trainees who tested positive (i.e., cases) for gonorrhea or chlamydia at Joint Base San Antonio–Lackland during any point in their BMT from 2017 through 2023. Additionally, during this study period, from November 2021 through March 2022, 352 male BMT trainees as well as active duty, reserve, and National Guard members were tested for gonorrhea and chlamydia as part of a previously published universal screening study that did not evaluate follow-up testing, so they were also included in this study. All male cases were matched 1-to-1 with female BMT trainees (i.e., controls) by age, date of military accession, and pathogen testing positive during training to determine sex-based differences in follow-up testing. ^8^ Urinary testing for gonorrhea and chlamydia was performed by nucleic acid amplification testing (Hologic, Marlborough, MA) throughout the entire study period. ^2^

For all positive male cases, a retrospective chart review in the Joint Legacy Viewer and MHS GENESIS electronic health records was performed. These systems include all military hospital and clinic records, regardless of geographic location. Variables including patient demographics, indications for testing, and testing facility were collected for each case. While current CDC guidelines recommend follow-up testing for re-infection at 3 months, this study evaluated whether a patient underwent repeat testing within 12 months of a positive test. ^2^

Chart reviews identified positive laboratory test results for gonorrhea and chlamydia in BMT trainees. Test results for 3 years after original gonorrhea or chlamydia diagnosis were reviewed, or until July 1, 2024 if a period of 3 years following original diagnosis had not elapsed by initiation of data collection, as that period of time was used for a previous study. ^9^

Nominal variables were compared by Fisher's Exact Test due to small sample size, and continuous variables were compared by a Mann-Whitney U test due to non-parametric data distribution. Standard odds ratios (ORs) with 95% confidence intervals (CIs) were also calculated. A *p* -value less than 0.05 was pre-determined to be statistically significant.

This study was reviewed by the 59th Medical Wing Human Protections Office and determined to be exempt from Institutional Review Board approval due to its retrospective nature, and thus, consent was not obtained from subjects.

## Results

Of the 182,726 male BMT trainees from 2017 through 2023, 50 active duty male trainees (0.03%) tested positive for gonorrhea or chlamydia during their 8 weeks of training (data not shown). Most cases (n=43, 86%) were due to chlamydia, with the remainder positive for gonorrhea **(Table 1)** . There were no cases of co-infection among male BMT trainees **(Table 1)** . During the same period, 5-6% of female trainees screened positive for chlamydia, and 0.2–0.4% screened positive for gonorrhea (data not shown).

**TABLE 1.** Description of Chlamydia and Gonorrhea Diagnoses, Testing and Symptomology Among 50 Male U.S. Air Force and Space Force Basic Trainees

	No.	%
Diagnosis		
Chlamydia	43	86
Gonorrhea	7	14
Both chlamydia and gonorrhea	0	0
Location of testing		
Primary Care	44	88
Emergency department	4	8
Occupational Health	1	2
Infectious Diseases	1	2
Indication for testing ^a^		
Symptoms	27	54
Screening	13	26
Contact	7	14
Symptoms and contact	3	6
Symptoms		
Dysuria	10	20
Dysuria and penile discharge	9	18
Penile discharge	6	12
Genital pain	3	6
Dysuria and penile vesicles	1	2
Groin pruritic	1	2
No symptoms	20	40
Training days elapsed until diagnosis		
	Median	IQR
	12.5	[8-27]

Abbreviations: No., number; IQR, interquartile range.

a Indication for testing exceeds number of patients, as some patients had multiple testing indications.

The median age of male BMT trainees was 20 years (IQR 19-21). Most cases (n=44, 88%) were detected in primary care settings, with a minority of cases (n=4, 8%) diagnosed in the emergency department. The median time in training until diagnosis was 12.5 days (IQR 8-27).

Thirty male trainees (60%) had symptoms on presentation for chlamydia and gonorrhea testing, with dysuria (n=20, 40%) and penile discharge (n=15, 30%) the most common **(Table 1)** . Nine (18%) male trainees were tested as part of the previously reported screening protocol, ^8^ while 10 (20%) were tested after being notified of STI exposure by a partner (data not shown). Four (8%) additional male BMT trainees were asymptomatically screened for chlamydia and gonorrhea after presenting to a medical provider for another medical problem, including 1 service member who tested positive during HIV screening (data not shown).

Of the male BMT trainees with chlamydia or gonorrhea, 28 (56%) had repeat testing in 1 year, with 5 testing positive for chlamydia and 1 for gonorrhea **(Table 2)** . Male trainees had statistically significant lower follow-up testing within 1 year compared to female trainees (56% vs. 76%; OR 0.41, 95% CI 0.17, 0.95) **(Table 2)** . Despite this difference in follow-up testing, there was no statistically significant difference in chlamydia and gonorrhea diagnoses during the next 3 years: a total of 8 diagnoses among men versus 12 among women (OR 0.6, 95% CI 0.22, 1.63) **(Table 2)** .

**TABLE 2.** Follow up Testing and Recurrence Among 50 Male U.S. Air Force and Space Force Basic Trainees with Matched Female Basic Trainees Diagnosed with Chlamydia or Gonorrhea, 2017–2023

		Males (n=50)		Females (n=50)					
	No.	%	No.	%	OR	CI (95%)
Follow-up within 1 year	28	56	38	76	0.41	0.17–0.95
STI within 3 years	8	16	12	24	0.60	0.22–1.63
Chlamydia	5	10	10	20	0.44	0.14–1.40
Gonorrhea	1	2	0	0	3.06	0.10–77.0
Both	2	4	2	4	1.00	0.14–7.40
None	42	84	38	76	1.66	0.61–4.49
HIV PrEP within 3 years	1	2	0	0	3.06	0.10–77.0

Abbreviations: No., number; OR, odds ratio; CI, confidence interval; STI, sexually transmitted infection; HIV, human immunodeficiency virus; PrEP, pre-exposure prophylaxis.

## Discussion

This retrospective matched cohort study evaluated 50 male Air Force and Space Force BMT trainees who tested positive for gonorrhea or chlamydia from 2017 through 2023. The majority of male BMTs who tested positive in this study presented for testing due to symptoms consistent with an STI. Only 56% of the men in this study received follow-up testing within 1 year.

When compared to prevalence rates of gonorrhea and chlamydia among the universally-screened female BMT population, the rate observed among the male BMT trainee population in this study is much lower than expected. When universally screened, 4.8% of male BMT trainees tested positive for chlamydia. ^8^ While the universal screening study included National Guard and reserve trainees in addition to active duty personnel, if that rate were applied to the population in this study, 8,771 cases of chlamydia would be expected among male BMT trainees. Given that only 43 cases of chlamydia were diagnosed in this study, it appears as though only 0.5% of expected cases of chlamydia were captured in this cohort. Notably, 9, or nearly 20%, of the cases in this study were identified through the previously published universal screening study. These results show that relying upon symptoms or partner notification likely missed thousands of infectious in the male BMT population.

Despite universal access to medical care, only 54% of male BMT trainees who tested positive for an STI in this study were re-tested within a year. CDC guidelines ^5^ ,11 recommend repeat testing in 3 months post-diagnosis due to the high risk of re-infection with the same or new STI pathogen. Similar to previous reports of follow-up testing in women in basic training, a relatively high positivity (18%) results on repeat testing. This finding suggests that a population with a bacterial STI who undergoes testing might be at greater risk for future infections in a male trainee population, and that there may be benefit from interventions such as Doxycycline Post-Exposure Prophylaxis (DoxyPEP), which has shown benefit in other populations, even decreasing incidence within a population.

There are several challenges related to STI testing in a military trainee population. First, due to the low reported incidence of STIs in BMT men, even if symptomatic, they are often not tested for bacterial STIs. Additionally, there is significant stigma related to STI positivity throughout the military that may be amplified in the BMT environment, the first stage of a service member's military career, during which trainees experience significant stressors unrelated to their sexual health. Other unique challenges within the military population can contribute to lower than ideal follow-up testing rates. The majority of BMT trainees are assigned to a different duty station after graduation, resulting in lack of continuity of care that likely contributes to diminished follow-up testing, although notably, female BMT trainees with gonorrhea or chlamydia who moved to a different military base evinced a higher follow-up rate than women who stayed on the base where they originally tested positive. Finally, military members often have a career-long focus on maintaining mission readiness, and preventive medical care, which can potentially change an individual's ‘mission ready’ status, is often avoided, as described in other military populations. ^13 , 18^

There are limitations to consider when interpreting these results. First, initial diagnoses and the start of data collection occurred within close temporal proximity. Although periods of time for follow-up testing were artificially shortened for some individuals, they should be similar for paired individuals, as matching was by accession date.

Second, the periods of service for men and women with chlamydia or gonorrhea may be different, which was not captured in this study and could lead to differences in observational time between men and women. Future studies could use person-time rates to adjust for varying follow-up durations.

In addition, patients empirically treated without testing were not captured, and the methodology did not allow ascertainment of the total number of male BMT trainees who tested negative for gonorrhea and chlamydia, and thus testing rates could not be determined.

This study did not evaluate extragenital testing, which has lower uptake compared to genital testing ^22^ and could have identified more individuals, resulting in more conservative estimates of infection.

Furthermore, the small sample of 50 men and 50 women may have limited this study's power to detect statistically significant differences between the 2 groups. Testing records before or after BMT for patients who did not test positive during the study period were not available for review, which likely contributed to an overall under-calculation of follow-up testing rates and new infection rates for both groups of BMT trainees.

There are potential benefits as well as drawbacks of implementing a universal STI screening program for male service members in the U.S. Air Force. While the true incidence of gonorrhea and chlamydia in this population is likely under-estimated due to asymptomatic infections and lack of routine screening, a screening program could identify individuals at risk and inform them of preventive health strategies. Furthermore, studies suggest that universal screening can be cost effective through the prevention of long-term health issues in female partners. Universal BMT male screening is not currently in place, however, due to the lack of long-term complications in men from untreated infections, its cost, and the administrative burden of testing. Despite these challenges, STI testing remains important for interrupting disease transmission, which has the potential to affect mission readiness through complications in female partners as well as increased HIV risk in both sexes.
